# Revolutionizing Diabetic Foot Ulcer Care: The Senotherapeutic Approach

**DOI:** 10.14336/AD.2024.0065

**Published:** 2024-04-16

**Authors:** Guiqin Zhang, Priyadarshani Nadeeshika Samarawickrama, Li Gui, Yuan Ma, Mei Cao, Hong Zhu, Wei Li, Honglin Yang, Kecheng Li, Yang Yang, Enfang Zhu, Wen Li, Yonghan He

**Affiliations:** ^1^Department of Endocrinology, the Second Affiliated Hospital of Dali University (the Third People's Hospital of Yunnan Province), Kunming, Yunnan 650011, China.; ^2^Key Laboratory of Genetic Evolution & Animal Models, Kunming Institute of Zoology, Chinese Academy of Sciences, Kunming, Yunnan 650201, China.; ^3^Key Laboratory of Healthy Aging Research of Yunnan Province, Kunming Institute of Zoology, Chinese Academy of Sciences, Kunming, Yunnan 650201, China.; ^4^Department of Orthopedics, the Third People’s Hospital of Yunnan Province, Kunming, Yunnan 650011, China.; ^5^Department of Biochemistry & Structural Biology, University of Texas Health Science Center, San Antonio, TX 78229, USA.

**Keywords:** cellular senescence, diabetic foot ulcer, senescent cells, senolytic, senomorphic, wound healing

## Abstract

Diabetic foot ulcers (DFUs) are a prevalent and profoundly debilitating complication that afflicts individuals with diabetes mellitus (DM). These ulcers are associated with substantial morbidity, recurrence rates, disability, and mortality, imposing substantial economic, psychological, and medical burdens. Timely detection and intervention can mitigate the morbidity and disparities linked to DFU. Nevertheless, current therapeutic approaches for DFU continue to grapple with multifaceted limitations. A growing body of evidence emphasizes the crucial role of cellular senescence in the pathogenesis of chronic wounds. Interventions that try to delay cellular senescence, eliminate senescent cells (SnCs), or suppress the senescence-associated secretory phenotype (SASP) have shown promise for helping chronic wounds to heal. In this context, targeting cellular senescence emerges as a novel therapeutic strategy for DFU. In this comprehensive review, we look at the pathology and treatment of DFU in a systematic way. We also explain the growing importance of investigating SnCs in DFU and highlight the great potential of senotherapeutics that target SnCs in DFU treatment. The development of efficacious and safe senotherapeutics represents a pioneering therapeutic approach aimed at enhancing the quality of life for individuals affected by DFU.

## 1. Introduction

Diabetes mellitus (DM) is a chronic condition characterized by persistent hyperglycemia that impacts multiple organ systems. As reported by the International Diabetes Federation in 2021, the global adult population afflicted by DM stands at approximately 537 million individuals, with a prevalence rate of 10.5%. This number is projected to surge to 783 million by the year 2045 [1]. Diabetic foot ulcers (DFUs) represent one of the gravest complications associated with DM, with its prevalence escalating in tandem with the rising incidence of DM.

**Figure 1.**
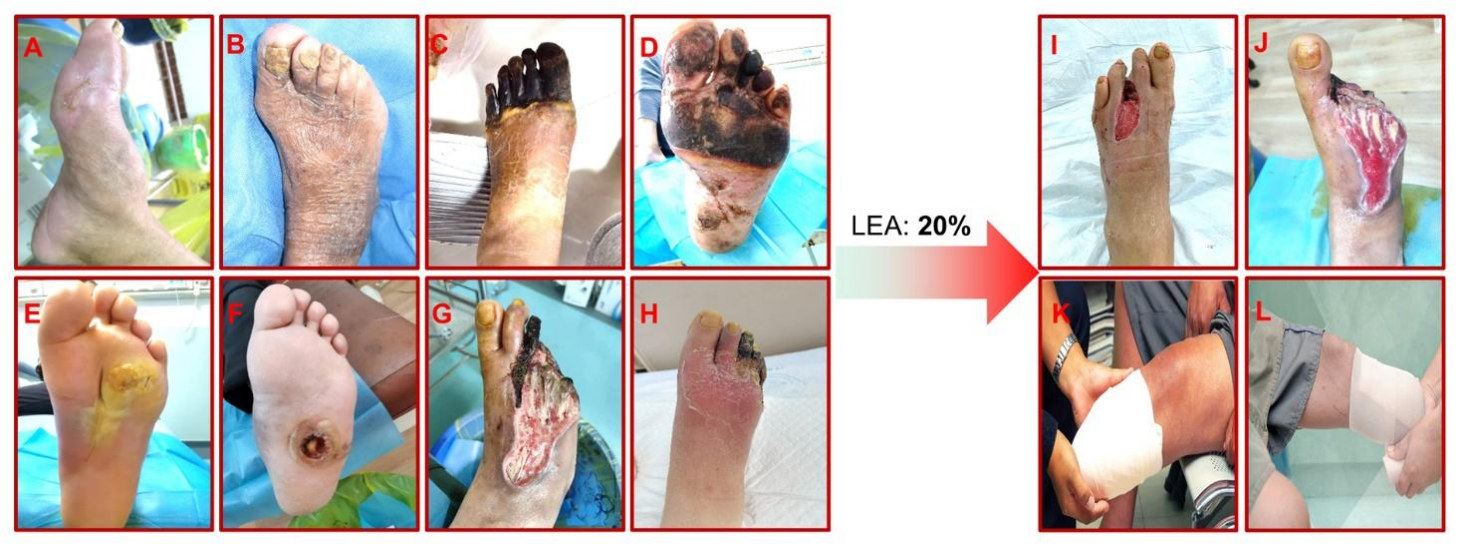
**Presentation of diabetic foot ulcers (DFUs)**. Characteristic cases of preulcerative (A and B), ischemic (C and D), neuropathic (E and F), and neuroischemic (G and H) DFU. 20% of DFU patients require minor lower-extremity amputation (LEA) (below the ankle, I and J), or major LEA (above the ankle, K and L). The DFU figures A-J are from the Third People's Hospital of Yunnan Province. K-L are from the network. The data on the prevalence, recurrence and mortality rate of DFU, as well as the mortality following amputations, are from the latest literature [7].

It has been reported that DFU affects approximately 6.3% of people with DM [2]. Alarmingly, around 20% of individuals who develop a DFU will ultimately require lower-extremity amputation (LEA) [3] (Fig. 1), and the five-year mortality rate following LEA surpasses 70% [4]. DM patients with DFU face a perilous five-year mortality risk that is 2.5 times higher than that of DM patients without DFU [5]. Moreover, hospitalization costs for DFU patients are a staggering 11 times higher than those for non-DFU patients [6]. Consequently, DFU imposes a substantial financial, emotional, and medical burden not only on patients but also on their families and society at large [7]. Furthermore, DFU is plagued by a daunting five-year recurrence rate that can reach up to 65%, constituting a primary driver of recurrent hospitalizations among DFU patients [3]. The long-term toxic effects of chronic hyperglycemia are what cause DFU and keep it coming back. This causes microvascular damage and nerve damage, which causes metabolic and nutritional problems in the skin of the affected foot [7]. Also, peripheral artery disease, peripheral neuropathy, infections, immune dysregulation, and metabolic dysfunction can slow or stop wounds from healing, which can lead to gangrene or the need for LEA [8, 9].

Current ways to treat DFU include controlling the level of glucose in the blood, improving microcirculation, providing neurotrophic support, preventing infections, optimizing nutrition, lowering plantar pressure, wound debridement, taking care of wounds and dressings carefully, re-building blood vessels, stimulating the spinal cord with electricity, and using multidisciplinary foot care teams, among other things. However, despite being helpful in reducing the rate of LEA and mortality among DFU patients [8, 10], the persistence of unfavorable outcomes, such as gangrene, LEA, and overall mortality, continues to pose a challenge.

Recent investigations [11, 12] have shed light on the involvement of cellular senescence in DFU, a factor contributing to delayed or non-healing wounds in these individuals. Hyperglycemia emerges as a pivotal player in the initiation of cellular senescence, concurrently precipitating oxidative stress, mitochondrial dysfunction, and DNA damage and instigating premature senescence across diverse cell types, including skin fibroblasts, endothelial cells, keratinocytes, and stem cells [11]. In addition, hyperglycemia fosters the generation of the senescence-associated secretory phenotype (SASP), culminating in compromised wound healing [11]. Conversely, interventions aimed at preventing cellular senescence or eradicating senescent cells (SnCs) have shown promise in promoting wound healing in the context of diabetes [13-16]. In light of these findings, targeting cellular senescence has emerged as a novel therapeutic approach for addressing DFU. Within this review, we have provided an exposition of the pathological underpinnings of DFU, with a particular emphasis on the emerging prominence of SnCs in this context. Also, we think that senotherapeutics, which include senolytics for selectively getting rid of SnCs and senomorphics for suppressing SASP, have a lot of therapeutic potential for treating DFU. The development of potent and safe senotherapeutics carries the promise of mitigating the morbidity and mortality associated with DFU.

## 2. Unveiling the presentation, etiology, and pathology of DFU

### 2.1 Etiology of DFU and wound pathologic features

**Figure 2.**
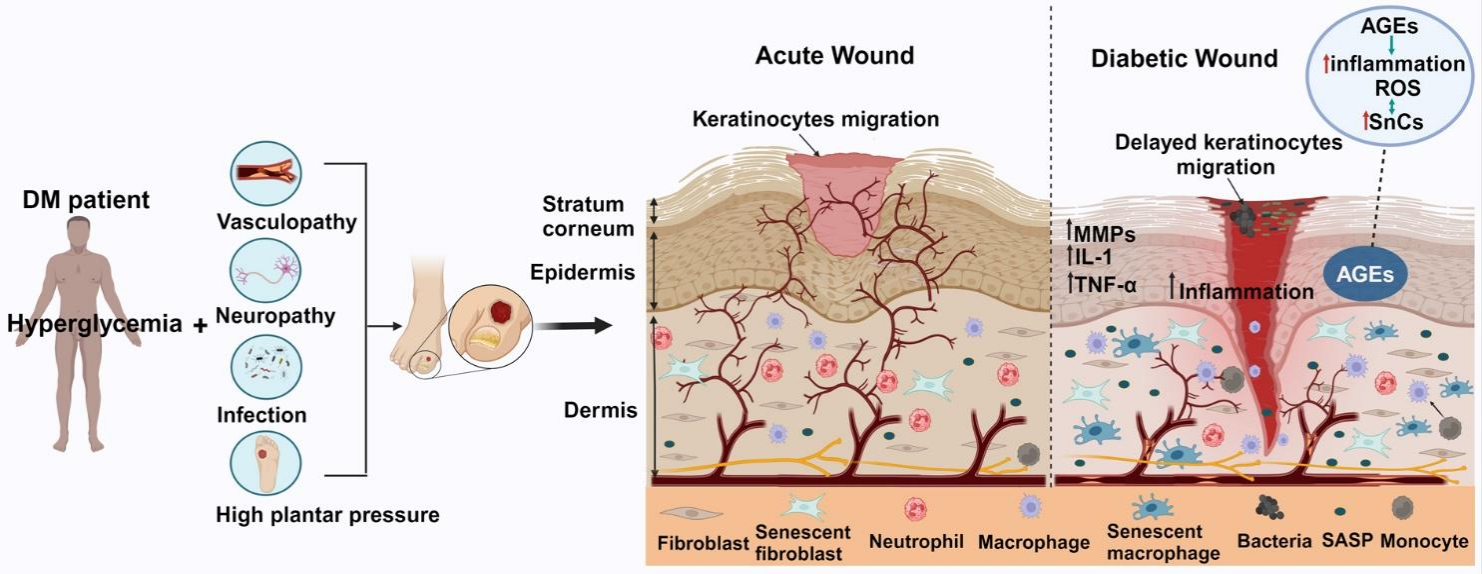
**Risk and pathology of acute wound and diabetic foot ulcer (DFU)**. Hyperglycemia, diabetic peripheral vasculopathy, peripheral neuropathy, infections, and elevated plantar pressure are key contributors to DFU. In the case of an acute wound, the inflammatory response is transient and fosters an environment conducive to wound healing. However, DFU belongs to the category of chronic wounds, characterized by a prolonged inflammatory phase with the formation of biofilm, impaired angiogenesis, and the onset of neurotrophic changes. The increasing number of senescent cells (SnCs) and the senescence-associated secretory phenotype (SASP) at the wound site may hamper the healing or DFU. AGEs, advanced glycation end products.

DFU manifests as a disruption in the epidermis and at least a portion of the dermis, primarily afflicting individuals with DM. It is classified as a type of chronic wound, alongside other chronic wound types like radiation ulcers, pressure ulcers, and vascular ulcers [17]. Like the healing process of other chronic cutaneous wounds, DFU wound healing is conventionally categorized into four sequential phases: hemostasis, inflammation, proliferation, and remodeling [18]. Wound healing is a complicated process that involves many different types of cells and growth factors. These include fibroblasts, platelets, macrophages, monocytes, neutrophils, endothelial cells, and keratinocytes, as well as important growth factors like fibroblast growth factor (FGF), platelet-derived growth factor (PDGF), transforming growth factor (TGF), vascular endothelial growth factor (VEGF), and epidermal growth factor (EGF) [12, 19]. The pathology of acute wounds and DFU is shown in Figure 2. Long-term hyperglycemia is toxic in a slow, insidious way that makes white blood cells less effective. This makes wounds more likely to get infected and to develop bacterial biofilms [12]. Moreover, chronic hyperglycemia inflicts damage on the microvascular system, ultimately contributing to arteriopathy and neuropathy in the lower extremities. Ischemia and hypoxia in the surrounding tissues are more proof that they play a major role in the development of chronic wounds in people with diabetes [5].

Pathologic features of chronic wounds include a buildup of SnCs, stem cells with incorrect function, high levels of pro-inflammatory cytokines, increased reactive oxygen species (ROS), and increased protease activity [17]. There are many factors that contribute to chronic wounds, and notably, aging and diabetes stand out as the two principal risk factors underpinning the development of chronic wounds [12], especially in DFU. The prevalence of DM, especially type 2 DM (T2DM), increases with age [20]. The cellular senescence contributes to the development of T2DM, and, in turn, the diabetic microenvironment provides favorable conditions for the induction of cellular senescence [21, 22]. Notably, senescent pancreatic β cells are a major cause of diabetes development and worsening [23]. Hyperglycemia causes a cascade of events, including oxidative stress, increased advanced glycation end products (AGEs), and ROS, ultimately inducing endothelial cell senescence [24, 25]. Endothelial cell senescence induces vascular structural and functional changes that enhance thrombosis, inflammation, and atherosclerosis [26, 27], leading to various diabetic complications, such as diabetic retinopathy, diabetic cardiovascular disease, diabetic nephropathy, and diabetic peripheral vasculopathy [21].

### 2.2 Pathogenesis of DFU

Diabetic peripheral vasculopathy assumes paramount significance as the predominant causative factor behind DFU. Atherosclerosis, or arterial occlusion, can precipitate microcirculatory disturbances within the feet stemming from inadequate blood perfusion. This impaired perfusion sets the stage for ischemic skin ulcers [28]. Furthermore, infection stands as a substantial contributor to the exacerbation and deterioration of DFU. Inadequate glycemic control exacerbates immune dysfunction, thereby compromising the skin's innate ability to ward off infections. Pathogens enter the foot's fascia, infiltrating deeper tissues like muscles, tendons, and even bones, when skin and soft tissues suffer compromise or microcirculation fails. This intrusion sparks infections, and when infections recur and necessitate prolonged broad-spectrum antibiotic treatments, the risk of developing multi-drug-resistant bacterial infections escalates, thereby increasing the likelihood of LEA among DFU patients [9]. Similarly, diabetic peripheral neuropathy and high plantar pressure play a pivotal role in the genesis of DFU. Autonomic neuropathy stops sweat glands in DFU patients' feet from making sweat, which could weaken the skin's protective barrier and make it more likely to get bacterial, viral, or fungal infections [29]. Damage to sensory nerves translates into abnormal sensations within the lower extremities of DFU patients. As protective sensations wane or vanish entirely, the foot's innate defense mechanism against environmental extremes such as cold, heat, and other injurious stimuli becomes compromised. Particularly, sensory neuropathy frequently co-occurs with the loss of skin integrity, rendering the foot's skin unresponsive to pressure, which, in turn, fosters prolonged and elevated plantar pressure. This sustained pressure precipitates mechanical damage to soft tissues [9, 30]. Furthermore, foot deformities, calluses, Charcot osteoarthropathy, and poorly fitting shoes can also lead to increased pressure in localized areas of the foot [8]. Motor neuropathy induces muscle and fat atrophy in the feet of individuals with DM, leading to altered plantar stress patterns and skin damage after extended periods of walking [8, 31]. All of the above factors work together and interact with each other, eventually culminating in the onset of DFU. Consequently, DFU wound healing remains a vexing challenge within clinical practice, giving rise to a myriad of complexities and obstacles in the realms of medical care and nursing intervention for individuals afflicted by DFU.

## 3. Clinical therapeutic strategy and challenges of DFU

### 3.1 Clinical challenges.

The challenging process of wound healing in DFU stands as a prominent clinical conundrum in the treatment of this condition. Factors such as diabetic peripheral vasculopathy, peripheral neuropathy, infections, various diabetic complications and comorbidities collectively exert profound influences on the healing trajectory of DFU wounds. Several key factors cause delayed-healing or non-healing ulcers, including narrowing or occlusion of lower extremity arteries, insufficient blood flow to the lower limb, tissue ischemia and hypoxia, nutritional imbalances, muscle atrophy in the foot skin, and slow growth of granulation tissue [32]. Also, diabetic peripheral neuropathy reduces or takes away the protective pain and temperature sensations in the foot. This increases the likelihood of re-injury or prolongs the healing process from an ulcer [8]. The compromised immune function of DFU patients heightens their vulnerability to infections. During the early stages of ulcer infections, hyperalgesia, often presenting as a negative symptom, may go unnoticed by patients and their families. Some infections can get deeper into the body even when broad-spectrum antibiotics are used during DFU management. This can lead to the formation of drug-resistant microbial biofilms, which can make the wound heal more slowly or not at all [33]. In instances where DFU patients concurrently grapple with chronic kidney disease or chronic heart failure, metabolic and nutritional disturbances further impede the healing process, contributing to delayed or non-healing ulcers [34].

### 3.2 Advancements and challenges in the current therapeutic approaches for DFU

Present-day interventions for DFU primarily encompass wound debridement, wound repair techniques, off-loading therapy, and negative pressure wound therapy, among others [8]. While the treatment landscape for DFU has expanded over time, the existing therapeutic approaches continue to grapple with certain limitations. In wound debridement treatments, physical debridement, which places high demands on the selection of an appropriate incision site and dilated area, remains the primary treatment modality for DFU [35]. Autolytic debridement may potentially exacerbate ulcer infections, culminating in sepsis, septicemia, or even LEA [36]. Enzymatic debridement necessitates extended cycle times and involves the use of costly materials [37]. Biological debridement, also known as maggot debridement, is somewhat less commonly employed in clinical practice due to the sensitivity and resistance exhibited by many patients towards maggots [38]. Wound repair is a widely acknowledged and vital therapeutic avenue for DFU. The application of biological engineering to damaged tissues places stringent requirements on the therapeutic process. Present transplantation techniques are limited to wounds displaying well-developed granulation tissue, an unobstructed blood supply, and a controlled infection [39]. Autologous platelet-rich gel, derived from a patient's whole blood through centrifugation, demands strict aseptic procedures during its preparation and application [40]. Wound biologics, including cytokines, are typically reserved for use on well-controlled ulcer surfaces that have completely removed necrotic tissue [41]. Hyperbaric oxygen therapy is employed to ameliorate tissue hypoxia, in spite of potential adverse effects such as middle ear pneumatic trauma, sinus/paranasal sinus pneumatic injury, pulmonary pneumatic trauma, and oxygen toxicity [42]. Stem cell transplantation technology, while promising, remains a subject of ongoing research, and its efficacy requires validation through further clinical investigations [43]. Off-loading therapy necessitates personalized decompression plans tailored to the patient's specific condition and their compliance with the recommended treatment regimen [3, 44]. Furthermore, DFU negative pressure wound therapy seeks to enhance wound healing by applying continuous or intermittent negative pressure within a sealed environment. Negative pressure suction does have some drawbacks, however. These include: 1) the risk of excessive blood loss in cases of active bleeding or vascular exposure; 2) the risk of making severe vascular stenosis or vascular occlusion worse; and 3) the risk of infection spreading inside the wound in cases of untreated osteomyelitis and septic arthritis [45]. Given the persistent challenges associated with the aforementioned treatments for DFU, there is an urgent need for novel and promising therapeutic strategies in clinical practice.

Recent years have witnessed a growing body of evidence highlighting the role of hyperglycemia in provoking oxidative stress, ultimately culminating in mitochondrial dysfunction and DNA damage, which in turn trigger cellular senescence. Simultaneously, hyperglycemia fosters the production of SASP, a factor that accelerates the onset of premature senescence across various cell types at the wound site, significantly compromising the healing process of the skin. As a consequence, the targeting of SnCs has emerged as a promising novel approach in the therapeutic arsenal against DFU. In the following sections, we will delve into the significance of this approach and its potential to revolutionize DFU treatment in clinical settings.

**Figure 3.**
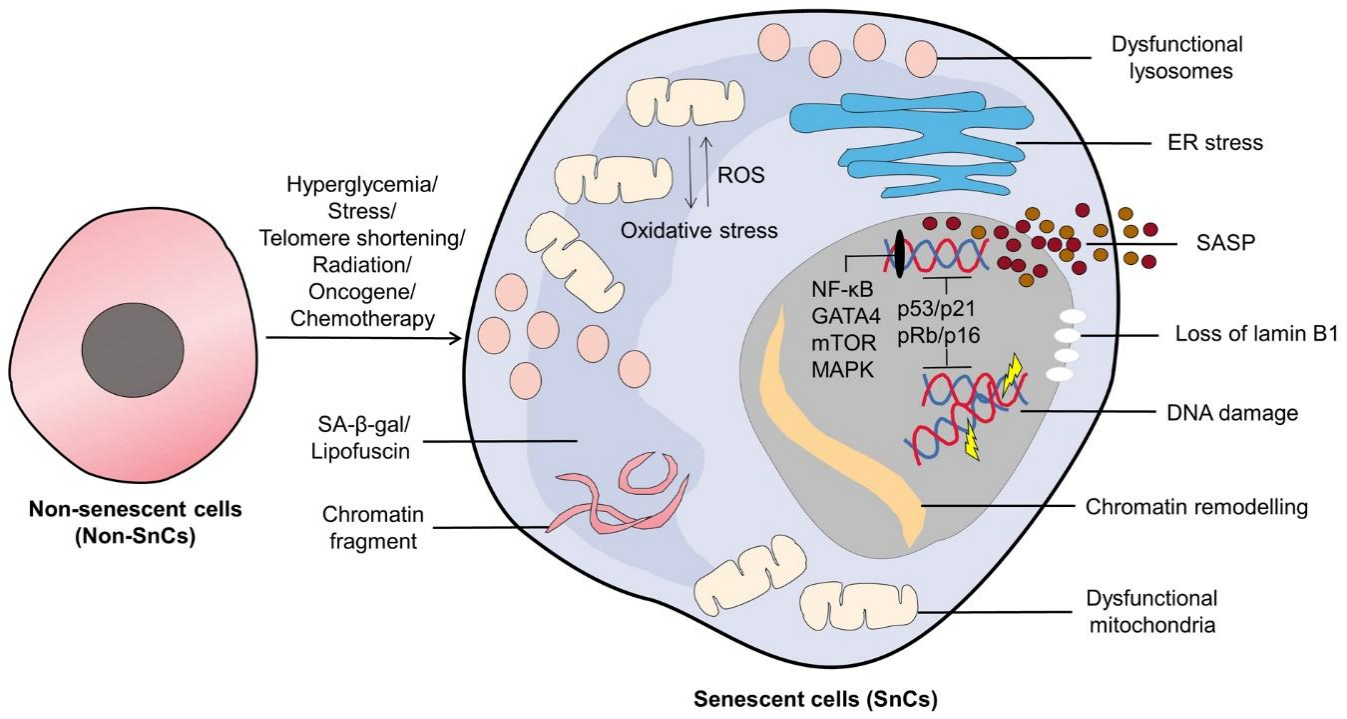
**Cellular senescence and hallmarks of senescent Cells (SnCs)**. Normal cells (Non-SnCs) undergo senescence in response to various stimuli. Senescent cells (SCs) exhibit distinctive characteristics, including cellular enlargement, DNA damage, heightened senescence-associated secretory phenotype (SASP), upregulation of cyclin-dependent kinase inhibitors p16 and p21, reduced lamin-B1 expression, accumulation of intracytoplasmic chromatin fragments, impaired mitochondrial and lysosomal function, and increased lysosomal β-galactosidase activity (SA-β-gal), etc.

## 4. Hallmarks and clinical significance of cellular senescence

### 4.1 Cellular senescence.

Leonard Hayflick and Paul Moorhead first noticed cellular senescence in 1961, when they were growing human primary fibroblasts in a continuous culture. This was a turning point in the field of cell biology [46]. These fibroblasts, initially exhibiting robust growth, eventually experience a progressive decline in their division and proliferation capacities, culminating in the loss of these vital functions and a subsequent decline in their population. This phenomenon was aptly termed 'cellular senescence' by Hayflick and Moorhead [46]. Subsequent research elucidated that this phenomenon is instigated by the gradual shortening of chromosomal telomeres and is recognized as telomerase-dependent replicative senescence (RS) [47], which has been shown to occur during aging of various types of human somatic cells in vitro and in vivo [48]. Additionally, various other stimuli have been identified as triggers for senescence, including persistent hyperglycemia, oxidative stress, activation of oncogenic genes, radiation, chemotherapeutic agents, and more [49, 50]. Notably, distinct stimuli give rise to specific types of cellular senescence, such as hyperglycemia-induced senescence, stress-induced premature senescence (SIPS), irradiation-induced senescence (IS), oncogene-induced senescence (OIS), and chemotherapy-induced senescence, among others [49, 50]. While the markers for senescence are neither uniform nor entirely specific, shared characteristics are observed in most cells undergoing senescence (Fig. 3) [49].

### 4.2 Hallmarks of cellular senescence

The characteristic feature of cell cycle arrest that distinguishes cellular senescence is its irreversible and permanent nature. In SnCs, this cell cycle stop is mostly caused by cyclin-dependent kinase (CDK) inhibitors called p21^WAF1/Cip1^ (p21) and p16^INK4a^ (p16). The oncogenes p53 and Rb also play a role, especially when p16 and p21 are more active or p16 and p21 are expressed more [51]. Activation of the DNA damage response (DDR) by senescence-inducing factors sets off a signaling cascade, orchestrated by p53, p21, and p16, which prevents cell cycle progression [52, 53]. Additional hallmarks of cellular senescence include enlarged cell size [54], diminished expression of lamin-B1 [55], formation of senescence-associated heterochromatin foci (SAHF) [56], metabolic disturbances, endoplasmic reticulum (ER) stress, mitochondrial and lysosomal dysfunction, etc. [57] (Fig. 3).

Importantly, SnCs actively produce a complex mixture of factors known as the SASP. These factors include inflammatory cytokines, chemokines, matrix metalloproteinases, growth factors, and insulin-like growth factor-binding proteins, among others [58-60]. The SASP is under the regulation of transcription factors such as nuclear factor κB (NF-κB), CCAAT/enhancer binding protein β (C/EBPβ), transcription factor GATA4 (GATA-binding protein 4), mammalian target of rapamycin (mTOR), and mitogen-activated protein kinases (MAPK) signaling pathways. It functions to induce inflammation, which in turn activates the immune response for the clearance of SnCs [61-64]. Senescence-associated β-galactosidase (SA-β-gal) activity is another characteristic feature of SnCs and is commonly used as a marker to identify cellular senescence [65]. The SASP contributes to the amplification and propagation of senescence by promoting paracrine and autocrine activities [66]. Notably, SnCs tend to develop resistance to apoptosis, a form of programmed cell death, as they accumulate in tissues [67]. This resistance to apoptosis results in the chronic secretion of SASP, which, in turn, causes damage to surrounding cells and tissues, contributing to inflammatory aging processes [60, 68, 69]. Under normal physiological conditions, SASP-induced transient aging triggers an immune response that gets rid of SnCs and encourages the growth and differentiation of progenitor cells [70, 71]. This helps damaged tissues heal. But in pathological situations, the continued release of SASP-mediated cellular senescence or a weaker immune system can cause SnCs to build up in tissues in an unusual manner. This can exacerbate tissue damage and diminish the repair capacity of damaged tissues, ultimately culminating in the formation of fibrosis [72].

In addition to the above features, cellular senescence per se is a highly heterogeneous process that is related to cellular origin and environmental stimuli [73]. There are different markers of senescence in different senescent tissues, and one marker may not be sufficient to define SnCs [74]. As mentioned earlier, DDR activation causes an increase in the level and activity of p53, which plays an important role in inducing cellular senescence. However, some cells undergo senescence that is accompanied by a decline in p53, such as keratinocytes and some human fibroblast cell lines. Thus, not all cells increase P53 expression during senescence [75]. Similarly, not all SnCs express high levels of p16; conversely, some cells with high p16 are not senescent (e.g., pancreatic β-cells, macrophages, endothelial cells) [74]. Analysis of bulk RNA sequencing (RNA-seq) data from different fibroblasts of humans and mice has shown differences in transcriptomic signatures and SASP based on senescence inducers, cell types, and stages of the senescence process [76]. Even targets that induce senescence in vitro with cell lines do not necessarily reflect the identity and function of pathogenic SnCs in vivo [77]. Therefore, the heterogeneity of SnCs is an important feature to consider when identifying SnCs.

### 4.3 Biological significance of cellular senescence

As previously described, cellular senescence is a phenomenon that occurs in both physiological and pathological processes, including tissue remodeling, injury, cancer, and aging. Whether cellular senescence has a beneficial or detrimental impact depends on the specific context, including the location and burden of SnCs [78]. Recent studies [21, 79-81] have shown that cellular senescence is associated with diseases such as T2DM, Alzheimer's disease, Parkinson's disease, and chronic obstructive pulmonary disease, among others [82]. In the short term, cellular senescence can have a positive effect on tissue remodeling and wound healing [83]. During the process of tissue repair, SnCs recruit immune cells to clear them and promote tissue renewal. However, when effective immune clearance cannot be achieved, the pathological effects of SnCs may gradually emerge as diseases progress [78]. In diabetic wounds, protracted activation of p53 induces cellular senescence by upregulating the cGAS-STING and NF-κB cascades and activating the p53-p21-Rb axis, impeding wound healing [84]. It is worth noting that p53 and p21 appear to be key regulators in DFU formation [85]. A recent study found that diabetic patients had significantly more p16 positive cells at the epidermal-dermal junction than non-diabetic patients after skin trauma or injury, suggesting that cellular senescence may be associated with the vulnerability of diabetic patients to chronic wounds [86]. In summary, senescence may have a beneficial role when activated in damaged cells exposed to acute stress, but it can be detrimental in the context of aging and chronic diseases when induced by chronic stress. In particular, cellular senescence promotes the occurrence and progression of diabetic ulcers and adversely affects chronic wound healing [87, 88] (Fig. 2).

## 5. Implications for DFU wound healing

Diabetic wound healing is subject to the influence of both local and systemic factors, often predisposed to develop into chronic wounds such as DFU. The elevated levels of inflammation and oxidative stress characteristic of chronic wounds create an environment conducive to the induction of cellular senescence [89]. Cellular senescence may play a big part in how DFU wounds heal, affecting how inflammation is controlled, how cells multiply, how blood vessels grow back, and how the extracellular matrix is reshaped (Fig. 2).

### 5.1 Cellular senescence in DFU wound healing.

According to the study by Demaria et al., skin damage can induce a transient state of senescence in fibroblasts and endothelial cells. SnCs, in turn, support the optimal healing of wounds by releasing PDGF-A, which stimulates the differentiation of myofibroblasts [83]. Interestingly, studies have shown that eliminating SnCs at this stage delays the healing of cutaneous wounds. The extracellular matrix (ECM) plays a crucial role in wound healing, serving as a framework for cell development and maintaining the structural integrity of damaged tissues [90, 91]. Upon wounding, the matricellular protein CCN1 (cellular communication network factor 1) binds to integrin α_6_β_1_ and the heparan sulfate proteoglycan (HSP), which causes ROS to be made and starts the DNA damage response at the wound site. This can induce fibroblast senescence by turning on pathways involving p53, p16, pRb, and p38 [90, 92, 93]. Senescent fibroblasts change the fibrotic response by increasing matrix metalloproteinases (MMPs). These MMPs break down the ECM and control how much of it is deposited [83, 90]. Therefore, cellular senescence plays a crucial role in promoting skin repair and regeneration during the early stages of wound healing while also regulating fibrosis and thus proving indispensable value for optimal wound healing.

Most of the time, immune surveillance by macrophages eliminates the temporary cellular senescence that wound healing causes [94]. Macrophages, during this phase, transition from a pro-inflammatory phenotype (M1) to a pro-healing phenotype (M2). M2 macrophages play a pivotal role in wound healing by promoting angiogenesis, fibroblast proliferation, and ECM remodeling. In order to do this, they make growth factors like TGF-β, VEGF, PDGF, and insulin-like growth factor-1 and lower inflammation by producing interleukin-1 receptor antagonists and other such molecules [95]. However, in the context of DM, wounds exhibit a shift in macrophage polarization towards the M1 pro-inflammatory phenotype and senescence, which is associated with aberrant activation of the cGAS-STING pathway [13, 96]. Furthermore, high blood glucose levels cause macrophages in diabetic wounds to activate NLRP3 inflammatory vesicles, which then split pro-caspase-1 into its active form, caspase-1. This active caspase-1 causes pro-inflammatory cytokines, like interleukin-1β (IL-1β), to be released. This keeps M1-type macrophages polarized and stops the formation of M2-type macrophages through positive feedback loops [97]. In addition, diabetic macrophages not only become less polarized toward the M2 phenotype, but they also have trouble phagocytic and chemotactic abilities, which makes SnCs build up in diabetic wounds [12, 98]. Research by Wilkinson et al. [13] has shown that SnCs accumulate in delayed-healing diabetic mouse wounds, with a significant portion of these SnCs being macrophages, which are more susceptible to senescence in diabetic mouse wounds compared to non-diabetic ones. Since macrophages are important immune cells that clear SnCs, their inability to function helps keep an inflammatory SASP going [17].

### 5.2 SASP in wound healing.

SnCs produce SASP, which has both positive and negative effects on various aspects of wound healing. SnCs recruit and activate immune cells through the secretion of chemokines (CCL2, CXCL2, and CXCL3) and inflammatory cytokines (IL-1β, IL-2, IL-6, and IL-8), which then contribute to the clearance of SnCs [99]. Notably, IL-6, IL-8, and the transcription factor C/EBPβ activate and amplify the inflammatory network, while IL-6 exerts a pro-inflammatory effect through paracrine secretion [100]. Furthermore, the proinflammatory chemokine IL-8/CXCL8 regulates endothelial cell migration and promotes angiogenesis [101]. SnCs also secrete pro-fibrogenic factors (such as TGF-β, IL-11, and PAI-1) that induce fibroblast activation and collagen deposition, while growth factors (including EGF and PDGF) influence the proliferation and differentiation of progenitor cells [99]. Additionally, angiogenesis was stimulated by secretion of VEGF in senescent fibroblasts [102], and the deposition of the ECM is regulated by the secretion of MMPs, which, in turn, stimulate epithelial cell proliferation [90, 103]. SnCs with high levels of pro-inflammatory and pro-fibrotic factors, however, are difficult for immune cells to remove from chronic wounds [99]. The increased protease activity in chronic wounds leads to the breakdown of dermal ECM, degradation of growth factors (such as VEGF and TGF-β), and inhibition of dermal reconstruction [104, 105]. Additionally, it has been observed that fibroblasts in chronic wounds exhibit reduced responsiveness to mitogen-stimulating growth factors (such as FGF, EGF, and PDGF) and impaired intracellular signaling [106].

**Figure 4.**
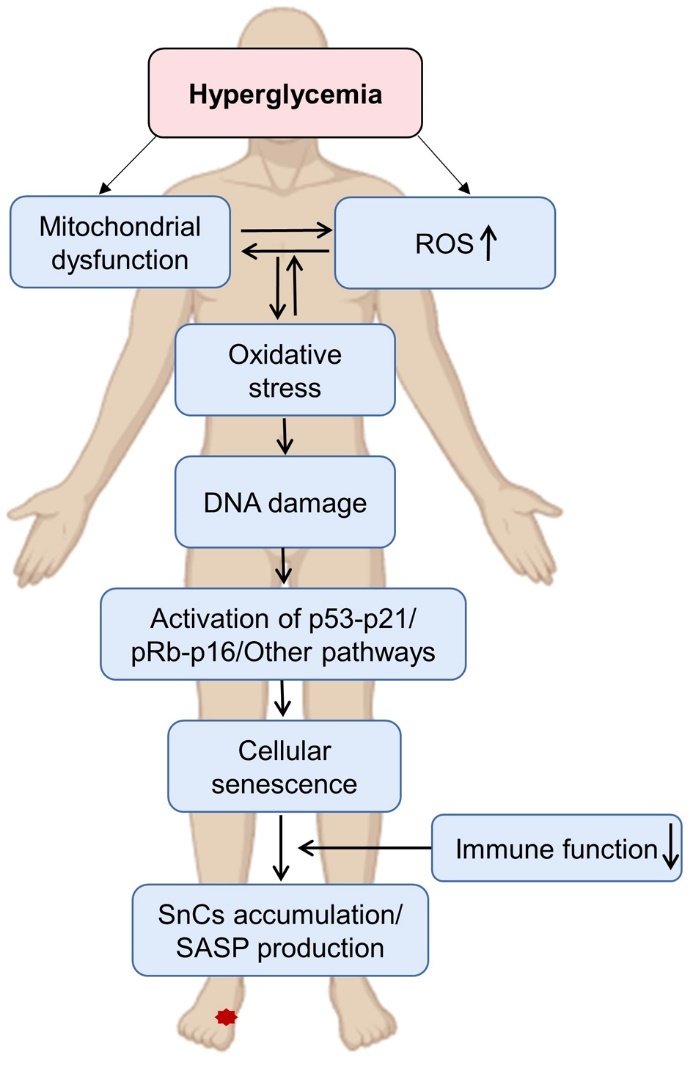
**Pathway of hyperglycemia-induced senescence in diabetes mellitus**. Hyperglycemia causes mitochondrial dysfunction and the production of reactive oxygen species (ROS) which induces DNA damage, and in turn activates the p53-p21, pRb-p16, and other pathways such as NF-κB to trigger cellular senescence. Hyperglycemia also exacerbates immune dysfunction, thereby compromising the ability to eliminate SnCs (SnCs).

Hyperglycemia plays a pivotal role in promoting cellular senescence and instigating various senescence responses (Fig. 4), including the secretion of the SASP [11]. Diabetic SASP contributes to the development of non-healing wounds by increasing insulin resistance, amplifying inflammation in intravascular and local tissues, transmitting senescence signals, and impairing ECM deposition in diabetic wounds [107, 108]. Notably, the SASP observed in chronic wounds like DFU is fundamentally pro-inflammatory and anti-proliferative, in stark contrast to the SASP associated with acute wounds [11]. Studies have demonstrated an upregulation in the expression of key SASP components such as CXCL1 and CXCL2, along with their co-receptor CXCR2, in diabetic mouse wounds. Intriguingly, CXCL2 induces senescence in human dermal fibroblasts through paracrine secretion mechanisms [13]. Moreover, hyperglycemia and oxidative stress induce senescence in fibroblasts, endothelial cells, and keratin-forming cells, resulting in the accumulation of SnCs at the site of wound repair following skin injury [17, 109, 110]. Persistently high blood sugar causes cellular senescence and speeds up the non-enzymatic glycosylation of the ECM, which makes advanced glycation end products (AGEs) [107, 111]. AGEs play a pivotal role in impaired wound healing by altering dermal structure, reducing the skin's ability to withstand mechanical stress, and binding to the receptor for AGEs (RAGE) to trigger oxidative stress, activate the NF-κB pathway, sustain inflammatory responses, and promote apoptosis [112, 113].

### 5.3 Cell stemness and plasticity in wound healing.

SASP induces cell stemness and plasticity, playing a crucial role in promoting tissue regeneration. Ritschka et al. have shown that when primary mouse keratinocytes are transiently exposed to SASP, genes related to stemness are activated to facilitate tissue regeneration. However, prolonged exposure to SASP can induce cell cycle arrest and senescence through paracrine signaling and inhibit regenerative processes [114]. Multiple stem cell niches exist in the epidermis of the skin, particularly those associated with hair follicles, and when the skin is injured, multiple cells can acquire stem cell properties to repair the damage [99]. Mesenchymal stem cells (MSCs) are multipotent stem cells that modulate the wound repair response by bringing in progenitor cells, fibroblasts, keratinocytes, and macrophages. Moreover, MSCs may induce angiogenesis through the paracrine secretion of cytokines, including VEGF, TGF-1, FGF, and IL-6 thereby promoting diabetic wound healing [115]. Interestingly, both pluripotent stem cells and progenitor cells exhibit senescence characteristics similar to those of other non-stem cells [99]. Consequently, dysfunctional stem cells may impede or slow down the process of chronic wound healing [116].

Premature senescence and apoptosis in MSCs can be attributed to three primary factors: hyperglycemia, oxidative stress, and mitochondrial dysfunction [117, 118]. Hyperglycemia induces an increase in ROS, which can damage mitochondrial DNA (mtDNA) and lead to mitochondrial dysfunction. This, in turn, leads to a further increase in ROS production, and ROS accumulation promotes MSC senescence by causing oxidative stress and activating the p53 pathway [117, 118]. A sustained hyperglycemic environment induces MSCs senescence, leading to MSCs dysfunction, including decreased cell proliferation and differentiation capacity, angiogenesis, and immunomodulatory capacity [119, 120]. Senescent MSCs not only secrete inflammatory SASP to exert their pro-inflammatory effects [121], but also induce premature senescence in neighboring young hematopoietic stem cells (HSCs) via paracrine action [122]. Additionally, hyperglycemia-induced factors, such as reduced hypoxia-inducible factor-1α (HIF-1α) transcriptional activity, decreased expression of VEGF and stromal cell-derived factor-1 (SDF-1), impair neovascularization and the functionality of endothelial progenitor cells (EPCs) [123, 124]. Consequently, the proliferation and migration of EPCs at the wound site in diabetic patients are reduced [116]. The healing trajectory of DFU is impinged by a self-expanding and self-perpetuative SnCs society that drives wound chronicity. This society may be fostered by diabetic SASP, negatively affecting DFU wound repair [11].

In summary, cellular senescence plays a dual role in the wound healing process. During the early stages of wound healing, cellular senescence promotes tissue repair and regeneration. However, as the healing process progresses, SnCs begin to exert adverse effects on the healing of chronic wounds. In essence, while physiological cellular senescence plays a beneficial role in acute wounds, preventing cellular senescence or selectively removing SnCs may prove advantageous for the treatment of chronic wounds. There is evidence that cellular senescence represents a novel and evolving therapeutic target with significant potential for enhancing DFU wound healing [125].

**Figure 5.**
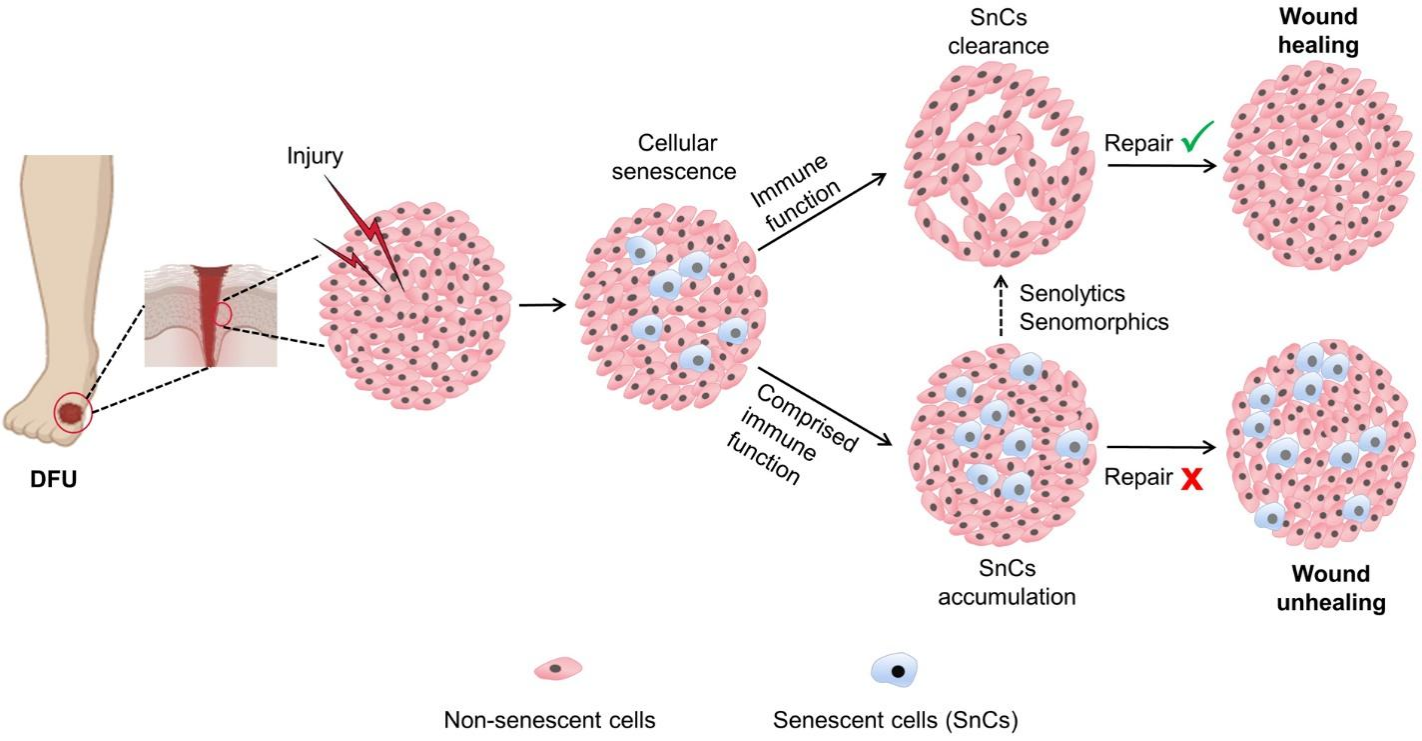
**The dual role of cellular senescence in wound healing**. Hyperglycemia together with major diabetes mellitus (DM) complications leads to wound of DFU. Cellular senescence exhibits a Janus-faced role in the context of wound healing. During the initial stages of acute wound healing, cellular senescence plays a beneficial role by promoting wound repair, with SnCs (SnCs) being efficiently cleared through immune surveillance. However, in chronic wounds like DFU, immune cells (i. e., macrophages) themselves undergo senescence, leading to impaired phagocytosis and chemotaxis. The resulting accumulation of SnCs further propagates senescence and exacerbates inflammation, contributing to non-healing wounds. The targeted use of senolytics or senomorphics holds potential for facilitating wound healing, e.g., in DFU.

## 6. Targeting SnCs as an emerging intervention for DFU

During the chronic stage of wound healing, the accumulation of SnCs at the wound site can impede the healing process (Fig. 5). Wilkinson et al. [13] have shown that macrophages in diabetic mice release SASP factors that are high in CXCL1 and CXCL2. These factors cause cellular senescence and slow wound healing. SB-265610, which is a CXCR2 antagonist, has been shown to reduce neutrophil infiltration and macrophage senescence. This can reverse delayed healing in diabetic mice and accelerate ex vivo human skin wound healing [13]. Extracellular vesicles (EVs) play an important role in wound healing as mediators of intercellular communication. A study by Wei et al. has revealed that plasma endothelial cells-derived-EVs prevent fibroblast senescence and accelerate skin wound healing in diabetic mice by reducing YAP phosphorylation and activating the PI3K/AKT/mTOR pathway [126]. Mesenchymal stem cell-derived small EVs mitigate oxidative stress-induced senescence in endothelial cell via regulation of miR-146a/Src, accelerating the healing of diabetic mouse wounds through increased angiogenesis [127]. The nicotinamide adenine dinucleotide (NAD)-dependent deacetylase sirtuin-1 (SIRT1), a member of the NAD-dependent enzyme family, has the capacity to inhibit transcription factors such as p53, NF-κB, P38MAPK, and MMP-9 [128]. Activating SIRT1 reduces oxidative stress, protecting blood vessels. SIRT1 agonists like resveratrol have been shown to stimulate angiogenesis and expedite wound healing in diabetic mice by mitigating hyperglycemia-induced endothelial dysfunction [14, 15]. SDF-1, a member of the chemokine family, plays a pivotal role in recruiting endothelial progenitor cells to the wound’s surface. However, hyperglycemia leads to reduced expression of SDF-1, inhibiting diabetic wound healing [123]. The senescence-associated factor dipeptidyl peptidase 4 (DDP4) is an exopeptidase that causes the loss of N-terminal dipeptides and proinflammatory effects [129]. When the dipeptidyl-peptidase-4 (DPP4) inhibitor MK0626 was given locally to diabetic mice, it was found to increase angiogenesis and SDF-1 expression at the wound site. This led to a greater number of bone marrow-derived mesenchymal progenitor cells (BM-MPCs) in diabetic wounds and sped up the overall diabetic wound healing process [16]. Upregulation of the cGAS-STING pathway in diabetic wounds hinders wound healing by exacerbating inflammation and senescence [96]. While using gene-editing to silence STING in macrophages in diabetic mice wound treatment by cell therapy can induce the polarization of wound macrophages from pro-inflammatory M1 to anti-inflammatory M2, promote angiogenesis, and induce collagen deposition to accelerate diabetic wound healing [130]. In addition, some new techniques are used in the senotherapeutic treatment of diabetic wounds. Nanospheres of Fe_3_O_4_ were encapsulated with galactose-modified poly (lactic-co-glycolic acid) (PLGA) and are designed to target SnCs through gal modification, which selectively releases Fe_3_O_4_ and triggers an iron-ion-mediated Fenton reaction to eliminate the SnCs, accelerating the healing of diabetic wounds in mice [131]. Similarly, Zhao et al. utilized poly-l-lysine/sodium alginate (PLS) modified with tarabostat (PT100) and encapsulated the PARP1 plasmid for delivery to target the DPP4 receptor, eliminating senescent fibroblasts and decreasing SASP to promote wound healing in diabetic mice [132]. A novel bioactive wound dressing by loading miR-17-5p-engineered small EVs (sEVs17-OE) in gelatin methacryloyl (GelMA) hydrogel can inhibit senescence of endothelial cells and fibroblasts markedly repress p21 expression and stimulate proliferation, thus facilitating wound healing in diabetic mice [133]. These findings underscore the potential benefits of inhibiting cellular senescence or reducing SnCs in promoting diabetic wound healing. Thus, senotherapeutics may hold promise for enhancing DFU recovery.

## 7. Potential senotherapeutics for DFU

### 7.1 Senolytics.

Clearing out SnCs through genetic or pharmaceutical means has been shown to help treat diseases like T2DM [134, 135], neurodegenerative disorders [136, 137], nonalcoholic fatty liver disease, and fibrosis [138, 139]. Senolytics are small molecules that target proteins in the senescence cell anti-apoptosis pathways (SCAPs) and can selectively kill SnCs. This is an exciting new development in this field [68, 73, 140]. Since 2015, multiple classes of senolytics have been developed, encompassing naturally occurring compounds and their derivatives [141], and targeted therapeutics [68, 73, 140]. Senolytics such as Dasatinib and Quercetin (D+Q), fisetin and navitoclax have shown the capability to selectively remove SnCs, including those induced by viruses. They have been found to mitigate COVID-19-reminiscent lung disease and reduce mortality rates in mice [142, 143]. Wang *et al*. have shown that D+Q can reduce DNA damage by selectively removing SnCs in mice and rats. This keeps tissue cells' ability to divide and helps ulcers caused by radiation heal faster [144]. Additionally, D+Q has been confirmed to improve physical dysfunction in aged mice, resulting in a 36% increase in survival rates and a 65% decrease in mortality [145]. As of now, some senolytics have entered clinical trials and have shown promising effects [146]. Particularly, one clinical trial proved D + Q reduces the burden of SnCs in human adipose tissue and skin epidermis. This clearance is evidenced by a reduction in the expression of senescence markers like p16 and p21, decreased SA-β-gal activity, and a reduction in the secretion of SASP factors such as IL-1α, IL-6, MMP-9, and MMP-12 [147]. The results of several other clinical trials have been shown in Table 2. These results suggest that the elimination of SnCs holds promise as a new approach to treating DFU. With the focus on cellular senescence and disease, more and more senescence targets have been identified (Table 1).

**Table 1:** Potential senolytics and senomorphics for treatment of diabetic foot ulcers (DFUs).

	Name	Target	Function	Ref.
**Senolytics**				
	Dasatinib+Quercetin	SnC anti-apoptosis pathways (SCAPs)	Induce apoptosis by targeting SCAPs	[164]
	Navitoclax (ABT-263), ABT-737	BCL-2/BCL-XL/BCL-W	Inhibit anti-apoptosis proteins in intrinsic apoptosis pathway	[174, 175]
	A1331852, A1155463, UBX-1325	BCL-XL	Inhibit anti-apoptosis protein Bcl-xL in intrinsic apoptosis pathway	[176, 177]
	17-DMAG, XL888 Geldanamycin, 17-AAG, Tanespimycin, Ganetespib	HSP90	Inhibit HSP90 activity and trigger apoptosis	[77, 178]
	UBX0101, RG7112 (RO5045337)	MDM2 / p53	Induce SnCs apoptosis by restoring p53 activity through inhibition of MDM2/p53 interaction	[179, 180]
	P5091, P22077	USP7	Induce SnCs apoptosis by promoting MDM2 ubiquitination and degradation of the ubiquitin-proteasome system	[75]
	FOXO4-p53 interfering peptide	FOXO4	Disrupt the FOXO4-p53 interaction, allowing p53 to translocate to the cytosol and inducing cell-intrinsic apoptosis	[181]
	Piperlongumine (and analogues)	OXR1	Induce the death of SnCs by directly binding to OXR1	[182]
	Ouabain, Digoxin, Ouabagenin, Bufalin, Oleandrin, K-Stropanthin, Strophanthidin, Proscillaridin A	Na^+^/K^+^ ATPase	Inhibit the Na^+^/K^+^ -ATPase, causing destabilization of the SnCs membrane potential and intracellular acidification, and mediate SnCs apoptosis by inducing the proapoptotic protein NOXA	[183-185]
	JQ1	BET	Inhibitor of BET proteins that recognize acetylated chromatin and regulate gene expression	[186]
	BPTES	GLS1	Inhibitor of GLS1, inhibit glutaminolysis and eliminate SnCs	[187]
	EF24	Multiple targets, e.g. BCL-2 family protein	Increase degradation of the anti-apoptotic BCL-2 family proteins	[188]
	Azithromycin	Autophagy and glycolysis	Eliminate SnCs by inducing autophagy and improving glycolytic activity	[189]
	Roxithromycin	NOX4	Kill SnCs by inhibition of NOX4 pathway	[190]
	BET family protein degrader	NHEJ and autophagy	Degrade BET proteins, attenuate NHEJ expression, and upregulate autophagic gene expression	[186]
	Fisetin	BCL-2, PI3K/AKT/mTOR, NF-κB and more	Eliminate SnCs and reduce SASP through inhibition of multiple pathways (e.g. BCL-2, PI3K/AKT/mTOR, NF-κB)	[165, 191]
	Panobinostat	HDAC	Inhibitor of HDAC, decrease expression of BCL-XL and increase acetylated H3	[192]
	Fenofibrate	PPARα	Up-regulate PPARα expression, reduce SnCs via apoptosis	[193]
	Tamatinib (R406)	FAK and p38MAPK	Syk inhibitor, induce SnCs apoptosis by inhibiting phosphorylation of FAK and p38MAPK	[194]
	MitoTam	Mitochondria	Selective elimination of SnCs by reducing mitochondrial membrane potential and inhibiting OXPHOS	[195]
	AT-406	IAP	Elimination of SnCs by inhibition of c-IAP1, c-IAP2 and XIAP	[196]
	Phloretin	Sodium/glucose cotransporters	Inhibitor of sodium/glucose cotransporters, decrease glucose uptake and increase apoptosis	[197]
	GL-V9	Autolysosome	Alkalize the lysosome and elevate ROS levels, aggravating mitophagy deficiency to induce apoptosis	[198]
	RSL3	GPX4	Reduce the expression of GPX4 protein, and selectively induce SnCs death by the induction of ferroptosis	[199]
	Nintedanib	JAK2/STAT3	Inhibition of the JAK2/STAT3 pathway to induce the selective death of SnCs	[200]
	S63845, UM177, AZD5991	Mcl-1	Inhibitors of Mcl-1, induce SnCs apoptosis by upregulation of cleaved caspase 3	[201]
	25-hydroxycholesterol	CRYAB	Induce SnCs death by specific down-regulation of CRYAB	[202]
	Nigericin	K^+^	Reduce the K^+^ content in SnCs and induce pyroptosis	[203]
	CUDC-907	HDAC/PI3K	Inhibitor of HDAC/PI3K, induce SnCs apoptosis by increasing p53 and reducing BH3 pro-survival proteins	[204]
	N-myristoyltransferase inhibitors (NMTi)	COPI	Inhibition of coatomer complex I (COPI) results in Golgi dispersal, dysfunctional autophagy, and unfolded protein response-dependent apoptosis of SnCs	[205]
	PF-04691502	PI3/AKT and mTOR	Inhibit the phosphorylation of S6K and AKT and induce apoptosis in SnCs	[206]
**Senomorphics**				
	Metformin	IKK and/or NF-κB	Suppress SASP by inhibiting the phosphorylation of IκB and IKKα/β to block the translocation of NF-κB to the nucleus	[207]
	Resveratrol and STACs, Dapagliflozin	SIRT1	Prevent cellular senescence and suppress SASP by activating SIRT1	[157, 208, 209]
	Cordycepin	AMPK, NRF2	Regulate AMPK and NRF2 activity	[152]
	Rapamycin, RAD001	mTOR	Inhibitor of mTOR kinase, suppress SASP	[210, 211]
	SB-265610	CXCR2	Inhibit macrophage senescence by blocking CXCR2	[13]
	DPP4 Inhibitors	AMPK/SIRT1/Nrf2, PKA	Inhibit cellular senescence by regulating AMPK/SIRT1/Nrf2 and/or PKA pathways	[212]
	NDGA	5LOX, TK	Inhibit 5LOX and TK and antioxidation	[213]
	Rutin	ATM, HIF1α and TRAF6	Restrain SASP by interfering with the interactions of ATM with HIF1α and TRAF6	[154]
	Apigenin, Kaempferol, BAY 11-7082	NF-κB p65 subunit and IκB	Inhibition of NF-κΒ pathway, suppress SASP	[214-216]
	SB203580, UR13756, BIRB796	p38 MAPK	Inhibition of p38 MAPK pathway, suppress SASP	[217, 218]
	(5 Z)-7-Oxozeaenol	TAK1	Inhibition of TAK1 led to reduced mTOR and SASP	[211]
	Ruxolitinib	JAK	Inhibitor of JAK pathway, suppress SASP	[219]
	KU-60019	ATM	Alleviate cellular senescence by recovering mitochondrial function in normal fibroblasts	[220]
	KU-55933	ATM	Downregulate NF-κB activation and reduce senescence and SASP expression	[221]
	Loperamide	HSP90, Ca^2+^	Ca^2+^ channel blockers, SASP suppression in ERCC1-/- MEFs (RS)	[178]
	Cortisol & Corticosterone	IL-1α / NF-κΒ	Inhibit IL-1α / NF-κΒ positive feedback loop, suppress SASP	[222]
	Telomeric antisense oligonucleotides	tncRNAs, DDR	Inhibit telomeric non-coding RNAs (tncRNAs) function, prevent DDR activation and premature cellular senescence	[223]
	NBD peptide	IKK	IKK inhibitor, delay age-related pathologies and suppress SASP	[224]
	SR12343	IKK/NF-κB	Inhibition of IKK/NF-κB activation, reduce senescence and SASP	[225]
	MK2.III, PF-3644022	MK2 kinase	Inhibition of p38MAPK downstream target effect MK2 kinase and inhibition of SASP secretion of SnCs	[218]
	Mmu-miR-2910-3p	TGF-β receptor 2	TGF-β receptor 2 inhibitor, reduce mRNA and protein expression of TGF-β receptor 2, p53, and p21	[226]
	Epigallocatechin gallate (EGCG)	p53	Suppression of p53 acetylation	[227]
	Genistein	mTOR, AMPK	Inhibit mTOR signalling and activate AMPK pathway	[228]
	Oleuropein aglycone, Hydroxytyrosol	NF-κB	Reduce SA-β-gal-positive cells and p16 protein expression, as well as attenuate markers of SASP	[229]
	Glucosamine	mTOR, p70S6K	Inhibit the phosphorylation of mTOR and p70S6K, activate autophagy	[230]
	Aspirin	Asymmetric dimethylarginine (ADMA)	Delay endothelial senescence by preventing a decrease in NO formation/generation.	[231]
	Loc14	PDIA3	Suppress SASP by inhibiting the expression of protein disulfide isomerase family A member 3 (PDIA3)	[232]
	Terreic acid	BTK, NF-κΒ	Inhibitor of BTK, improve mitochondrial function and ATP production via OXPHOS, and inhibit NF-κΒ pathway and SASP	[233]
	Y-27632	ROCK	Inhibitor of Rho-associated protein kinase (ROCK) reduces the expression of NF-κB effector genes and IκBζ downstream mediators	[234]
	Daidzein metabolite 6,7,4'-THIF	PKCα	Inhibition of PKCα by 6,7,4'-trihydroxyisoflavone (6,7,4'-THIF) reduces UV-induced MAPK signaling and MMP-1 expression	[235]
	U0126	MEK/mTOR	Suppress senescence by inhibiting the MEK/ mTOR pathway	[236]
	Methylphenidate	PP2A	Activator of PP2A, attenuate the DDR and reduce SASP gene expression and SA-β-gal	[237]
	Trametinib	mRNA splicing factor	Adjust the level of mRNA splicing factor and attenuate senescence phenotypes	[238]
	Melatonin	mTORC1, MMP-1	Reduce the aging-promoting mTORC1 activity and MMP-1 expression	[239]
	2,5-dimethylpyrazine (DMP)	TRPM5	A natural pheromone TRPM5 ion channel activator, mitigates the spontaneous ageing-associated suppression of epidermal proliferation	[240]
	Ginsenoside Rg1	Wnt/β-Catenin, SIRT1/ FOXO3, SIRT3/SOD2, SIRT6/NF-κB and more	Inhibit oxidative stress, DNA damage, and cell cycle arrest by regulating multiple pathways	[241]
	Pyruvate dehydrogenase kinase 4 (PDK4)	YAP and JNK	Improve the senescent phenotype through the enhancement of glycolysis and regulation of YAP and JNK pathway	[242]
	n-butylidenephthalide	Nrf-2/HO-1	Suppress senescence and decrease ROS production via activating Nrf-2/HO-1 pathway	[243]
**Compounds with both senolytic and senomorphic activity**				
	Procyanidin C1	N.A.	At low concentration, the expression of SASP is inhibited, and at high concentration, SnCs may be selectively killed by promoting production of ROS and mitochondrial dysfunction	[159]
	Gingerenone A	Caspase-3	Induce SnCs death and decrease SASP by increasing the pro-apoptotic protein caspase-3 cleavage	[160]
	Solidago virgaurea subsp. alpestris	PRT, JAK/STAT, mTOR/PI3K/AKT and more	Reverse "papillary to reticular transition" (PRT) and reduce SASP, and induce SnCs apoptosis through short-term treatment with high doses	[162]
	Isatis tinctoria extract (active components not analyzed)	Caspase-3	Induce caspase-3-dependent apoptosis in SnCs as well as suppress the SASP at the post-transcriptional level	[161]
	Curcumin, o-Vanillin (curcumin metabolite)	AKT, NRF2, NF-κB	Inhibition of AKT and NRF2 pathways mediate apoptosis, reduce SASP through the down-regulation of the NF-κB	[244]
	Lipophilic Statins	HMG-CoA reductase, Mevalonic acid	HMG-CoA reductase inhibitor, inhibits oxidative stress and SASP, eliminates SnCs through inhibition of the mevalonate pathway	[245, 246]
**Other strategies**				
	SSK1, Pro-drug A (JHB75B), Nav-Gal, 5FURGal	SA-β-gal, Lysosomal activity of SnCs	Increased SA-β-gal activity, leading to the release of the cytotoxic compound and selective killing of SnCs	[171, 172, 247, 248]
	PZ15227	BCL-XL	A PROTAC that degrades BCL-XL	[181]
	ARV825, ARV771	BRD4	A PROTAC that degrades BRD4	[186, 249]
	CAR-T	uPAR	Selectively target SnCs expressing urokinase-type plasminogen activator receptor (uPAR) and enhance immune clearance of SnCs	[138, 169, 170]
	CRISPR/Cas9 or AONs	PTBP1	Suppress SASP by inhibiting the expression of PTBP1	[250]
	Magrolimab	CD47	Block the interaction between CD47 and SIRPα to enhance macrophage clearance of SnCs	[251]
	Immune checkpoint blockade (ICB)	PD-L1	Selective inhibition of accumulation of senescent PD-L1^+^ cells by ICB	[252]
	Antibody-drug conjugates (ADCs)	B2M	ADCs against β2-microglobulin (B2M) clears SnCs by releasing cytotoxic compound	[253]
	Nanocarriers	Senescence markers, e.g. SA-β-gal	As a targeted drug delivery vehicle for SnCs, and protect the drug from premature degradation	[254, 255]
	Toxin-conjugated anti-human CD2 antibody (hCD2-SAP)	hCD2, p16INK4a	Eliminate p16-positive cells, including senescence-associated T cells, and ameliorate age-associated phenotypes of CD4^+^ T cells	[256]
	Mitochondria-ablating agent. Mito-K2	Mitochondria, Aryldithiol-containing peptide oligomerization	Oligomerization and self-assembly disrupt the mitochondrial membrane, inducing mitochondrial dysfunction and selectively eliminating SnC	[257]
	GPNMB vaccine	GPNMB	Eliminate SnCs through antibody-dependent cellular cytotoxicity (ADCC) by targeting glycoprotein nonmetastatic melanoma protein B (GPNMB).	[258]

### 7.2 Senomorphics.

Instead of actively getting rid of SnCs, some naturally occurring compounds can either stop stressed cells from going into a senescent state or stop SnCs from making SASP factors. These compounds are referred to as senomorphics and have been comprehensively reviewed elsewhere [148, 149]. One such example is resveratrol, which has been shown to protect human endothelial cells and fibroblasts from stress-induced senescence [150, 151]. Cordycepin, a natural nucleoside analogue, has been reported to prevent radiation-induced ulcers by inhibiting cellular senescence through modulation of AMPK and nuclear factor erythroid 2-related factor 2 (NRF2) in rodents [152]. Additionally, cordycepin improves lysosomal function and encourages the restoration of autophagy levels in SnCs, which further inhibits stress-induced cellular senescence [153]. Recent research by Liu et al. has highlighted rutin as a potent senomorphic agent with the potential to target SnCs and enhance the efficacy of chemotherapy in mice [154]. Many chemical compounds also exhibit significant senomorphic activities. For example, rapamycin stops mTOR from functioning, which increases the expression of mitochondrial superoxide dismutase (MnSOD), decreases the release of SASP factors, and improves the function of keratinocytes. This leads to the suppression of epithelial stem cell senescence, ultimately reducing radiation-induced mucositis and ulcer formation in mice [155]. Drugs used to treat diabetes such as metformin and dapagliflozin may also offer health benefits through their senomorphic effects [156, 157]. In a mouse model of chronic muscle damage, turning off the endocytic bridging protein Numb causes myofibroblasts to age in a way that depends on p53. This makes it harder for muscles to heal [158]. However, p53 ablation and antioxidant treatment have been shown to reduce muscle senescence and restore the regenerative capacity of Numb mutants [158]. A variety of senomorphics are shown in Table 1. These studies provide valuable insights into the potential development of senomorphics for the treatment of DFU.

### 7.3 Compounds with both senolytic and senomorphic activities.

Some compounds can have both senolytic and senotherapeutic effects (Table 1). For instance, the flavonoid procyanidin C1 can inhibit SASP formation at lower concentrations while selectively eliminating SnCs in a wide spectrum of cell types and stressors at higher concentrations [159]. Gingerenone A, a novel senolytic/senomorphic, induces the death of senescent human fibroblasts and decreases the secreted levels of the pro-inflammatory factors IL-6, CCL2 (MCP-1) and interferon γ-induced protein 10 (IP-10) by increasing the pro-apoptotic protein caspase-3 cleavage [160]. Similarly, Isatis tinctoria, woad extracts, cause caspase-3-dependent apoptosis in senescent human skin fibroblasts as well as suppressing the SASP at the post-transcriptional level [161]. An extract from the plant Solidago virgaurea subsp. Alpestris can reduce the expression of various SASP components, ameliorating the negative influence on nearby cells. However, it exhibits moderate senolytic activity by inducing apoptosis in senescent human dermal fibroblasts at short-term high doses [162]. DFU is characterized by SnCs accumulation and high levels of inflammation. This class of compounds appears to exert a more comprehensive senotherapeutic effect to promote diabetic wound healing. Notably, some compounds still need more research to support their senotherapeutic effects.

**Table 2:** Currently published clinical studies on senotherapeutics.

Name	Study design	Sample size	Key findings	Ref.
**D + Q**	An open label Phase I pilot study	N=9	D + Q treatment significantly reduced SnCs burden in humans within 11 days.	[147]
**D + Q**	An open-label, proof-of-concept, phase I clinical study	N=5	Central nervous system penetrance of D was observed with outcomes supporting safety, tolerability and feasibility in patients with Alzheimer’s disease.	[259]
**D + Q**	A two-center, open-label, phase I clinical study	N=14	One serious adverse event was reported, and non-serious events were primarily mild-moderate. D + Q treatment may alleviate physical dysfunction in idiopathic pulmonary fibrosis (IPF).	[260]
**D + Q**	A phase I, single-blind, single-center, randomized, placebo-controlled pilot trial	N=12	There were no serious adverse events related to D + Q. Intermittently dosed D + Q in patients with IPF is feasible and generally well-tolerated.	[261]
**D**	A multicenter, single-arm, open-label Phase II trial	N=12	A decrease expression of SASP and other senescence -related gene sets in the skin of systemic sclerosis patients was associated with D treatment and correlated with clinical improvement.	[262]
**UBX1325**	A multicenter phase I clinical trial	N=8	Treatment with UBX1325 is well-tolerated and has the potential to lead to long-lasting disease modification for diabetic macular edema.	[263]

### 7.4 New strategies targeting SnCs.

Senotherapeutics have demonstrated their potential for treating age-related diseases. However, due to the heterogeneity of SnCs, senescence effects are not universal but are specific to particular cell types or specific senescence triggers [163]. For example, dasatinib exerts potent senolytic effects on fat cell progenitors, while quercetin better targets endothelial cells [164]. Both fisetin and navitoclax eliminate senescent fibroblasts and endothelial cells but have limited senolytic effects on senescent preadipocytes [165, 166]. Furthermore, they often come with on-target and off-target toxicities that restrict their clinical utility. In particular, targeting BCL-XL by navitoclax causes thrombocytopenia in humans [167]. To enhance their effectiveness while minimizing side effects, novel strategies for SnCs have emerged. These innovative approaches encompass proteolysis-targeting chimera (PROTAC) technology [168], T cell engineering [138, 169, 170], and β-galactosidase-targeted prodrugs [171, 172]. As an illustration of this progress, we have effectively utilized PROTAC technology to mitigate the on-target toxicity of navitoclax on platelets. This was achieved by transforming navitoclax into PZ15227, a BCL-XL specific PROTAC [168]. Further details on alternative methods for SnCs targeting can be found in our prior review [173]. Moreover, other new strategies have been organized in Table 1.

## 8. Conclusions and perspectives

DFU is a prevalent and severe complication of diabetes, and its pathogenesis involves intricate and multifaceted pathways [7]. Despite significant advancements in current therapeutic approaches that have improved the quality of life for DFU patients, there remain several limitations, as discussed earlier. Chronic wounds caused by DFU are in part caused by cellular senescence. Targeting SnCs with senolytic or senomorphic interventions could be a new way to treat DFU. In addition, combining anti-infection treatments with senotherapeutics has the potential to facilitate the healing process. Before senotherapeutics can be applied to the treatment of DFU, there are several important considerations. Firstly, it is crucial to conduct in-depth investigations into the role and mechanisms by which SnCs contribute to DFU wound healing [76]. The dynamic and highly heterogeneous nature of the senescence program necessitates a comprehensive understanding of how SnCs influence the course of DFU. This knowledge will be instrumental in developing new senotherapeutic strategies tailored to DFU. Secondly, it's important to acknowledge that currently identified senotherapeutics may have certain drawbacks, including limited potency or the presence of on-target and off-target toxicities. Given the vulnerability and compromised immune function of DFU patients, the development of potent and safe senotherapeutics is imperative. Lastly, alongside advancements in senotherapeutic approaches, there is a pressing need for large-scale improvements in preventive care and foot screening to reduce the incidence of DFU and lower-extremity amputations. With emerging efficacy data from clinical trials focused on senotherapeutics, the prospect of employing senotherapeutic strategies for the treatment of DFU appears promising and could become a reality in the near future.
